# Uptake of severe acute respiratory syndrome coronavirus 2 spike protein mediated by angiotensin converting enzyme 2 and ganglioside in human cerebrovascular cells

**DOI:** 10.3389/fnins.2023.1117845

**Published:** 2023-02-16

**Authors:** Conor McQuaid, Alexander Solorzano, Ian Dickerson, Rashid Deane

**Affiliations:** Department of Neuroscience, Del Monte Institute Neuroscience, University of Rochester, University of Rochester Medical Center (URMC), Rochester, NY, United States

**Keywords:** brain endothelial cells, brain pericytes, brain vascular smooth muscle cells, COVID-19, SARS-CoV-2 variants of interest and lipid raft

## Abstract

**Introduction:**

There is clinical evidence of neurological manifestations in coronavirus disease-19 (COVID-19). However, it is unclear whether differences in severe acute respiratory syndrome coronavirus 2 (SARS-CoV-2)/spike protein (SP) uptake by cells of the cerebrovasculature contribute to significant viral uptake to cause these symptoms.

**Methods:**

Since the initial step in viral invasion is binding/uptake, we used fluorescently labeled wild type and mutant SARS-CoV-2/SP to study this process. Three cerebrovascular cell types were used (endothelial cells, pericytes, and vascular smooth muscle cells), *in vitro*.

**Results:**

There was differential SARS-CoV-2/SP uptake by these cell types. Endothelial cells had the least uptake, which may limit SARS-CoV-2 uptake into brain from blood. Uptake was time and concentration dependent, and mediated by angiotensin converting enzyme 2 receptor (ACE2), and ganglioside (mono-sialotetrahexasylganglioside, GM1) that is predominantly expressed in the central nervous system and the cerebrovasculature. SARS-CoV-2/SPs with mutation sites, N501Y, E484K, and D614G, as seen in variants of interest, were also differentially taken up by these cell types. There was greater uptake compared to that of the wild type SARS-CoV-2/SP, but neutralization with anti-ACE2 or anti-GM1 antibodies was less effective.

**Conclusion:**

The data suggested that in addition to ACE2, gangliosides are also an important entry point of SARS-CoV-2/SP into these cells. Since SARS-CoV-2/SP binding/uptake is the initial step in the viral penetration into cells, a longer exposure and higher titer are required for significant uptake into the normal brain. Gangliosides, including GM1, could be an additional potential SARS-CoV-2 and therapeutic target at the cerebrovasculature.

## Introduction

While severe acute respiratory syndrome coronavirus 2 (SARS-CoV-2) primarily elicits respiratory infectious coronavirus disease-19 (COVID-19) (Bchetnia et al., 2020), many non-respiratory organs are also affected, including the brain (Huang et al., 2020; Mao L. et al., 2020; Moriguchi et al., 2020; Saleki et al., 2020; Brady et al., 2021; McQuaid et al., 2021), heart (Dhakal et al., 2020; Puntmann et al., 2020; Perez-Bermejo et al., 2021), kidneys (Fanelli et al., 2020; Martinez-Rojas et al., 2020), and liver (Mao R. et al., 2020; Wang et al., 2020; Zhong et al., 2020; Marjot et al., 2021). This may suggest that there is also viral distribution from the blood during the pathogenesis of this disease. There is continuous evolution of SARS-CoV-2 variants that affect people of all ages, which may lead to short- and long-term symptoms, including neurological manifestations. Several cell types associated with the neurovasculature are likely to interact with SARS-CoV-2, which may modulate/restrict its entry into the parenchyma. Thus, three cell types of the human cerebrovasculature (endothelium, pericytes, and smooth muscle cells) were used to determine if there were differential mechanisms of SARS-CoV-2 uptake. The data will provide a mechanistic basis for further studies, and hopefully contribute to the development of targeted therapeutic approaches, especially for “long COVID-19.”

The spike protein (SP) of SARS-CoV-2 is a structural protein, which is assemble as a trimer of the heterodimer (S1-S2), that protrudes from the viral surface to give it the crown-like appearance (Gordon et al., 2020; Li et al., 2020). The S1 unit contains a receptor binding domain (RBD), which promotes attachment to host cells *via* facilitators, such as to the extracellular peptidase domain on angiotensin converting enzyme 2 receptor (ACE2), the main receptor for SARS-CoV-2 (Hamming et al., 2004; Doobay et al., 2007; Hoffmann et al., 2020; Perrotta et al., 2020; Ma et al., 2021). TMPRESS 2 (transmembrane protease, serine 2) on the host cells cleaves the SP to promote viral entry into cells (Matsuyama et al., 2020). There are other receptors/facilitators on the cell surface that mediate the entry of SARS-CoV-2, including oligosaccharide receptors *via* sialic acid (Chen et al., 2005; Tortorici et al., 2019; Erickson et al., 2021; Rhea et al., 2021). Thus, there are multiple interaction sites between SARS-CoV-2 and host cells, which may contribute to cell type specific effects and the diverse symptoms of COVID-19 (McQuaid et al., 2021).

SARS-CoV-2 has been detected in brains of severely infected deceased people, however, it is unclear as to how it gets there and if this leads to significant viral neuro-invasion (McQuaid et al., 2021). Recombinant spike proteins have been used to study viral behavior by using *in vitro* models of brain endothelial cells and *in vivo* studies (Buzhdygan et al., 2020; Brady et al., 2021; Rhea et al., 2021). While it was reported that SP interacts with the brain endothelial cells, this was independent of ACE2, in mice (Rhea et al., 2021). The entry of SARS-CoV-2 into brain would be influenced by its interaction with several cell types, including the endothelium at the interface between blood and brain, and pericytes and smooth muscles cells. However, SARS-CoV-2/SP uptake by these cell types has not been fully characterized. Thus, this study was necessary to provide fundamental basic scientific data on SARS-CoV-2 interaction at the cerebrovasculature so as to provide a better understanding to the field and for further studied.

Herein, we used fluorescently labeled SP of wild type (WT) and mutants (from variants of concern) to establish the mechanism of SARS-CoV-2/SP uptake by human cerebrovascular cells (endothelial cells, pericytes and smooth muscle cells). We show that there was differential SARS-CoV-2/SP uptake by these three cell types, with the endothelial cells showing the lowest capacity for the uptake, which will limit entry into the parenchyma. SARS-CoV-2/SP uptake was mediated by ACE2 and a ganglioside (mono-sialotetrahexasylganglioside (GM1). SARS-CoV-2/SPs with mutation sites N501Y and E484K and D614G, showed a higher uptake compared to control wild type SARS-CoV-2/SP, except for D614G in pericytes. The striking differences for these mutants were greater binding to sialic acid *via* wheat germ agglutinin (WGA) and neutralization of the mutant uptake was less effective than that of the wild type SARS-CoV-2/SP using anti-ACE2 or anti-GM1 antibodies. The added value of our findings to the existing evidence is to provide data showing that GM1, which is expressed at the cerebrovasculature, is likely also an entry point for SARS-CoV-2 into these human cells. This also brings SARS-CoV-2 close to ACE2, which likely facilitates its entry into host cells, since both ACE2 and gangliosides are mainly located in the lipid raft/caveolin. Also, our data have shown that even though SARS-CoV-2 uptake mechanisms are similar, there are subtle differences, which could contribute the differences in the infection outcomes.

## Materials and methods

### Materials

SARS-CoV-2 Spike proteins [recombinant SARS-CoV-2 Spike Protein (SP-RBD, Arg319-Phe 541; cat# RP-87678, HEK293 cell expressed and binds ACE2] was obtained from Life Technologies Corporation, Carlsbad, CA, USA. Mutants SPs and its control wild type protein were obtained from RayBiotech Inc., Peachtree Corners, GA, USA. Recombinant mutants N501Y (cat# 230-30184, expressed region Arg319-Phe541), D614G (Cat# 230-3030186, expressed region Arg319-Gln690), E484K (cat# 230-30188, expressed region Arg319-Phe541) and their control wild type SP (Cat# 230-30162, expressed region Arg319-Phe541) were also HEK 293 expressed. All SPs were labeled separately with Alexa Fluor 555, using a kit (Microscale protein labeling kit; ThermoFisher Scientific; Waltham, MA, USA) and by following the manufacturer instructions. Anti-ACE2 antibody (R&D Systems, Cat# AF933) was labeled with Alexa Fluor 488 by following the manufacturer instructions (Microscale protein labeling kit; ThermoFisher Scientific). In addition, the labeled SPs or anti-ACE2 antibody were purified using 3 kDa molecular weight cut-off ultrafiltration filter (Amicon Ultra Centrifugal Filter, Millipore). There was no detectable dye in the filtrate.

Antibodies raised against the extracellular domain of potential SP binding receptors were used. These include, anti-ACE2 antibody (R&D Systems, Cat# AF933) used at a low (10 μg/ml) and high (60 μg/ml) concentration; anti-TMPRSSE2 antibody (Invitrogen, Cat# PA5-14264) used at 13 μg/ml; anti-CD147 antibody (Invitrogen, Cat# 34-5600) used at 2.5 μg/ml; anti-NP-1 antibody (Invitrogen, Cat# PA5-47027) used at 2 μg/ml and anti-GM1 antibody (Abcam, Cat# Ab23943) used at 5 μg/ml. The concentration used was obtained from the manufacturer guidance. Wheat Germ agglutinin (WGA; Cat# L9640) and heparin (cat# H3393) were obtained from Sigma, and used at 10 and 100 μg/mL, respectively. Transferrin from human serum conjugated to Alexa Fluor 488 (Cat# T13342), BODIPY FL C5-Lactosylceramide complex to BSA (Cat# B34402) and BODIPY FL ganglioside (Cat# B13950) were obtained from ThermoFisher Scientific) and used at 10 μg/ml. Nystatin (Cat# J62486.09), and chlorpromazine (Cat# J63659) were obtained from ThermoFisher Scientific, and used at 25 and 10 μg/ml, respectively. Angiotensin II (cat# 1158/5) was obtained from R&D Systems and used at 0.1 μg/mL.

### Cell culture

Three cell types were selected to focus more on the vascular mechanisms of SARS-CoV-2/SP uptake since they are also present in the vasculature of other organs. Human Cerebral Microvascular Endothelial Cells (hCMEC/D3) were purchased from Millipore (#SCC066) and expanded in EBM-2 Endothelial Cell Growth Basal Medium (Lonza #00190860) with EGM-2 MV* Microvascular Endothelial Singlequot kit (Lonza #CC-4147) supplemented media. hCMEC/D3 cells were expanded in T25 flask (ThermoFisher Scientific #156367) on a collagen IV (50 μg/ml Sigma-Aldrich #122-20) based growth matrix. *hCMEC/D3 cells were cultured in modified EBM-2 MV medium (Lonza) containing (v/v) 0.025% VEGF, IGF and EGF, 0.1% bFGF, 0.1% rhFGF, 0.1% gentamycin, 0.1% ascorbic acid, 0.04% hydrocortisone, and 1% 100 U/ml penicillin, and 100 μg/ml streptomycin. When hCMEC/D3 cells were grown on glass slides (ibidi u-chamber 12 well glass slides #81201) for experiments, collagen and fibronectin (50 μg/ml Millipore FC014) were used as matrix. Human Brain Vascular Smooth Muscle Cells (HBVSMCs) were purchased from Sciencell Research Laboratories, Inc., Carlsbad, CA, USA (Cat#1100) and expanded in Smooth Muscle Cell Medium (Sciencell #1101) by following manufacturer’s guidelines. HBVSMCs cell were expanded in T25 flasks with a Poly-L-Lysine [PLL (0.01% Sigma-Aldrich #P4707)] based growth matrix. When HBVSMCs were grown on glass slides (ibidi u-chamber 12 well glass slides) for experiments, fibronectin (50 μg/ml Millipore FC014) was used as matrix. Human Brain Vascular Pericytes (HBVPs) were purchased from Sciencell (#1200) and expanded in Human Pericyte Cell Medium (Sciencell #1201) by following manufacturer’s guidelines. HBVP cell were expanded in T25 flasks with a PLL (0.01% Sigma-Aldrich #P4707) based growth matrix. When HBVP were grown on glass slides (ibidi u-chamber 12 well glass slides) for experiments, fibronectin (50 μg/ml Millipore FC014) was used as matrix. All cell types were split at 90% confluency into fresh matrix coated flasks or slides for growth and experiments. Medium was changed within 24 h of initial split and every 2–3 days thereafter. Cells were kept in an incubator (37°C, 5% CO*2* humidified) during growth and experiments. Studies at 4°C were performed in a fridge.

### Uptake experiments

Cells were grown to confluent on glass-wells slides (ibidi u-chamber 12-well glass slides), media removed, cells washed 3x with Hank’s Balance Salt Solution containing calcium and magnesium HBSS + Ca/Mg, glucose and bicarbonate [Gibco, Waltham, MA, USA (14025-092)] before exposure to SP diluted in HBSS + Ca/Mg at a given concentration (usually 100 nM) and kept in an incubator (37°C, 5% CO*2* humidified) for the duration of the experiment. At the end of the experiment, cells were washed with HBSS + Ca/Mg, fixed in 4% paraformaldehyde (PFA) for 10 min and mounted with ProLong™ Glass Antifade Mountant with NucBlue™ Stain (ThermoFisher Scientific P36985).

### Inhibition studies

Cells were pre-incubate with the inhibitor (diluted in HBSS) for 15 min and in the present of SP (100 nM) for 4 h at 37°C and 5% CO*2* in the humidified incubator. All cells were then washed, fixed, mounted, and imaged. Values were calculated as percentage of controls, which were SP uptake without the inhibitor but with the vehicle solution.

### Low temperature studies

Cells were pre-incubate in the fridge (4°C) for 30 min to adapt the cells to this temperature before running the experiment for 1 h in the fridge in the presence of SP-555 (100 nM). For comparison, cells were incubated in the incubator for 30 min followed by 1 h in the presence of SP-555 (100 nM). All cells were then washed, fixed, mounted and imaged. The person performing the experiments was blinded to the type of tracer used in the experiments.

### Immunocytochemistry (ICC) and imaging

Cells were grown to confluency on glass slides, medium removed, washed with HBSS + Ca/Mg and fixed in 4% PFA for 15 min. Cells were not permeabilized. The same primary antibodies used for inhibition assay were used in the ICC and at the same concentration. The secondary antibodies, which were conjugated to Alexa Fluor 488, were donkey anti-rabbit (Thermofisher Scientific #A32790), anti-goat (Thermofisher Scientific #A32814) and anti-mouse (Thermofisher Scientific #A32766), and used at 1:200 dilution. Cells were mounted using with ProLong™ Glass Antifade Mountant with NucBlue™ Stain (ThermoFisher Scientific P36985) and imaged on Axiovert 25 Zeiss Inverted microscope (Obrekochen, Germany).

### ACE2 binding to SP

Recombinant human ACE2 (HEC 293 cells; cat# 230-30165), RayBiotech Life, 663276971Inc663276971RCRevathi Chandrasekar663276971-944626917Please note that the bracket is missing in some occurrences in the article, hence either removed or included based on the sentence formation. Kindly check and correct if necessary. (Peachtree Corners, GA), dissolved in carbonate/bicarbonate buffer, was immobilized (2 μg/ml) on glass slides for 1 h at room temperature (RT), blocked with a non-protein buffer (Pierce Blocking buffer), washed, incubated with SP-555 at different concentrations in HBSS for 1 h at RT, washed, mounted, and imaged. SP-555 intensity at each concentration were analyzed and expressed as intensity/μm^2^. Values are mean ± SEM.

### Imaging and analysis

**FIGURE 1:**
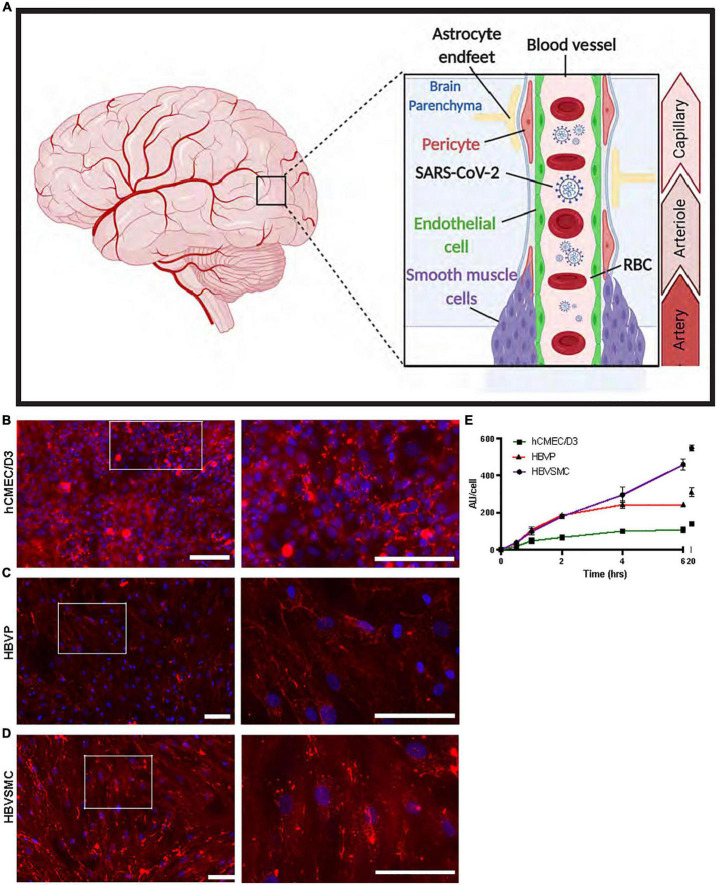
Progressive increase in SP uptake by cerebrovascular cells over time. **(A)** Schematic diagram showing the location of the three human cerebrovascular cell types used in this study [micro-vessel/capillaries endothelial cells (hCMEC/D3), pericytes (HBVP) and vascular smooth muscle cells (HBVSMC)]. Created by using BioRender.com. **(B–D)** Representative images of SARS-CoV-2 spike protein (SP)-555 (red) uptake, at 4 h, counter stained with 4’,6-diamidino-2-phenylindole (DAPI). Images on the right are the white boxed areas. **(E)** SP-555 uptake over time for the hCMEC/D3 (green), HBVP (red) and HBVSMC (purple). AU = Fluorescence arbitrary unit. Values are mean ± SEM. *N* = 3 to 6 wells per group. Scale bar = 100 μm.

**FIGURE 2:**
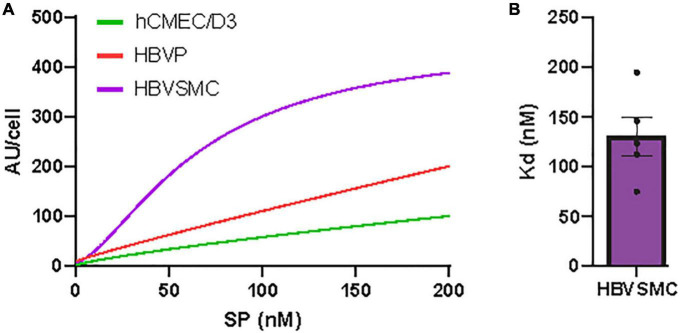
Concentration-dependent SARS-CoV-2/SP uptake by cerebrovascular cells. **(A)** SP-555 uptake at different SP-555 concentration for the hCMEC/D3 (green), HBVP (red), and HBVSMC (purple) using curve fitting. **(B)** Binding constant (Kd) for HBVSMC that was best fitted to a saturable uptake. There was no saturation for the other cell types over the concentrations used. Values are mean ± SEM. *N* = 3–5 wells/group Au = Fluorescence arbitrary unit. Supplementary Figures 1, 2.

Whole well images were taken on an Olympus VS-120 slide scanner. Exposure and gain were fixed for all experimental groups based on pilot experiments. All fluorescence (SP-555 and DAPI) quantification was performed without enhancement of signals. The person performing the analysis were blinded to the experimental design and tracer used. The VSI files were imported into Qupath for analysis. Five square fields with an area of 1 × 10^–6^ μm were randomly placed on each imaged. Custom pixel classifiers were created to measure the fluorescence intensity in the red (555 nm) channel. The settings for the pixel classifiers were standardized to be: random trees, moderate pixel resolution, and local mean subtraction set at 1. The cell count was performed with Qupath’s cell counter, the settings varied amongst the different cell types. Custom object classifiers for cell detection were created to correct the count of cells in the blue (359 nm) channel. Custom pixel threshold was used for analysis with controls on each slide and for each cell type. The analysis process was automated using scripts generated by Java Groovy in Qupath. Quantification of co-localization was done without enhancement and with custom pixel classifiers trained per slide within Qupath. Training image for the red channel, green channel, and merged channel were created, and subsequently a custom pixel classifier was created and trained per channel to perform the measurements. The five square fields were randomly placed on each imaged. The averaged area measurement was divided by its cell count to get AU per cell and averaged per well for standardization. Each slide had a control non-stained well to ensure proper training and analysis. The analysis process was automated using scripts generated by Java Groovy in Qupath. Data were expressed as fluorescence intensity per cell for standardization. Data for Figures 1, 2 were obtained using images from an Axiovert 25 Zeiss Inverted microscope (Obrekochen, Germany). These were not compared with any of the other data.

### Cell viability

Unlabeled wild type SP (0–200 nM) was used to measure *in vitro* cell viability by the MTT [3-(4,5-dimethylthiazol-2-yl)-2,5-diphenyltetrazolium bromide] assay [Roche, Cell proliferation Kit 1 (MTT) Cat no. 11465007001]. This is a colorimetric method according to which a tetrazolium-based compound is reduced to formazan by living cells. The amount of formazan produced is directly proportional to the number of living cells in the culture. Each cell type was incubated for 24 h at 37°C in the presence of 5% CO*2*.

### Statistic

All analysis were performed using Graphpad Prims version 9.2.0. Statistically analyzed was by analysis of variance (ANOVA) followed by Tukey *post-hoc* test for three or more groups. Unpaired *t*-test was used to compare two groups. The differences were considered to be significant at *p* < 0.05. For statistical representation, *P < 0.05, **P < 0.01, and ***P < 0.001 and ****P < 0.0001 are the levels of statistical significance. All values were expressed as mean ± SEM. *N* is the number of cell culture wells per group. For each well the average intensity of the five fields was used. Outliers were identified and removed using ROUT method with *Q* = 10% in GraphPad Prism version 9.2.0.

## Results

### Progressive SARS-CoV-2/SP uptake by cerebrovascular cells over time

The human brain cerebrovascular cells, endothelial cell (hCMEC/D3), primary pericyte (HBVP), and primary vascular smooth muscle cell (HBVSMC) were used (Figure 1A), as a monolayer to characterize the uptake mechanisms of wild type (WT) spike proteins (SP-555). While SP-555 signal was associated with each cell type, it was mostly seen on the cell surface of hCMEC/D3, but for the other two cell types there were more internalization (Figures 1B–D). The time-dependent uptake pattern, determined at 100 nM SP-555 and over 20 h, showed that the cell type approached equilibrium after 6 h with the exception of HBVSMC, which was linear (Figure 1E). The a half-time (t1/2) for equilibration was 2–3 h. The endothelial cells had the lowest capacity to take up SP compared to the other two cell types, possibly due to difference in the cell size and restricted uptake. SP-555 intensity values were expressed as per cell.

### Concentration-dependent SARS-CoV-2/SP uptake by cerebrovascular cells

The concentration-dependent SP-555 uptake was determined at 4 h, and this showed a pattern of approaching saturation after about 200 nM for the HBVSMC, while for the hCMEC/D3 and HBVP it was almost linear (Figure 2A). Higher concentrations were not used as previous studies used lower or about 100 nM (Buzhdygan et al., 2020; Rhea et al., 2021) and the relevant of higher concentration maybe questionable. The estimated binding affinity for the HBVMC was 100 nM (Figure 2B). However, this Kd value was greater than that for ACE2/SP-555 binding (protein-protein binding), *in vitro*, using a non-cellar assay (Supplementary Figure 1), which was similar to that of the manufacturer value that also used a non-cell-based assay. Thus, possible mechanisms of SP uptake are likely similar for these cell types but may be due to differential uptake by multiple facilitators. We confirm that SP was not toxic to these cell types (Supplementary Figure 2). Even though there are limitations in using the 3-(4,5-dimethylthazol-2yl)-2,5-diphenyl tetrazolium bromide (MTT) assay (Ghasemi et al., 2021), we used the same conditions for each cell type and followed the manufacturer instructions. This assay is dependent on the mitochondria in viable cells to metabolism and reduce MTT to formazan, a water-soluble violet-blue compound. Thus, the increased levels seen at the higher SP concentration for the hCMEC/D3 may be due to increased metabolic activity due to the higher mitochondria content of cerebral endothelium (Oldendorf and Brown, 1975; Oldendorf et al., 1977; Andrade Silva et al., 2021; Mullen et al., 2021). This increase in intensity is not due to toxicity since normally, toxicity is associated with a reduction in the intensity (lower mitochondrial activity and less viable cells). SARS-CoV-2 may cause lipid toxicity, as reported for HEK293 cells (Nguyen V. et al., 2022). This should not significantly affect our data since the experiments were performed at or less than 4 h, while the MTT assay was performed after 24 h incubation with the SP.

### ACE2 mediates SARS-CoV-2/SP uptake in cerebrovascular cells

**FIGURE 3:**
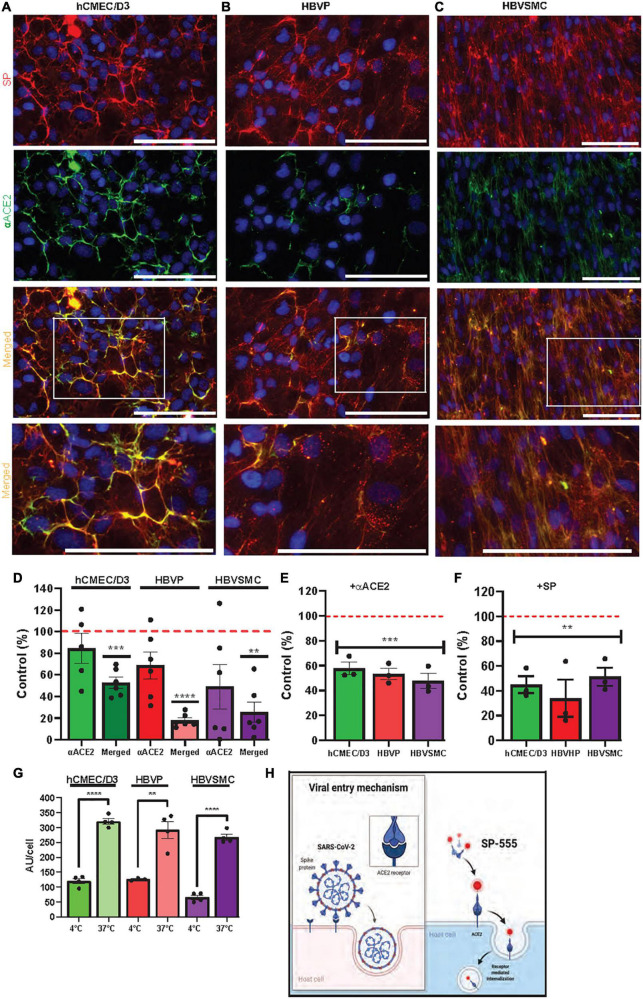
ACE2 mediates SARS-CoV-2/SP uptake in cerebrovascular cells**. (A–C)** SP-555 and anti-human ACE2 antibody (αACE2-488) co-localized on each of the cell types, hCMEC/D3 **(A)**, HBVP **(B)**, and HBVSMC **(C)**. Lowest row is the magnified image of the white boxed area above. **(D)** Intensities of αACE2 and merged SP/αACE2 (yellow) were expressed as a percentage of SP-555 AU (controls were the intensities of SP-555 cellular binding (all values were corrected as per cell). **(E)** Excess unlabeled αACE2 displaced SP-555 binding, which confirms the data in panel **(D)**. **(F)** Excess unlabeled SP (self-competition) displaced SP-555 uptake. Controls were the intensities of SP-555 cellular binding without excess unlabeled αACE2 (panel **E**) or unlabeled SP (panel **F**). **(G)** SP-555 receptor binding (4°C) considerable less than that at 37°C. **(H)** Schematic diagram showing that SP-555 mimics SARS-CoV-2 binding to ACE2. Adapted from “Proposed Therapeutic Treatments for COVID-19 Targeting Viral Entry Mechanism by www.biorender.com (2021). Retrieved from https://app.biorender.com/biorender-templates. Red dashed line is the control levels (100%). Values are mean ± SEM. *N* = number of well use (each data point). Statistically analyzed was by analysis of variance (ANOVA) followed by Tukey *post-hoc* test. *P < 0.05, **P < 0.01, and ***P < 0.001 and ****P < 0.0001. GraphPad Prism version 9.2.0 was used. Scale bar = 100 μm. Supplementary Figures 1, 3.

The pattern of SP uptake indicates some receptor binding and these cell types express a number of receptors that are associated with SARS-CoV-2, such as ACE2, a major binding site of SP (Supplementary Figure 3). ACE2 is expressed in human brain endothelial cells (Qiao et al., 2020), and in pericytes and vascular smooth muscle cells (He et al., 2020). We confirmed that ACE2 interacts with SP-555, *in vitro*, by using a non-cell-based assay (Supplementary Figure 1). The levels of ACE2 detected with an anti-ACE2 antibody (αACE2-488;10 μg/ml) were similar for the hCMEC/D3, HBVP and HBVSMC cells, and this represented about 50 to 80% of SP-555 cellular binding (Figures 3A–C). Thus, ACE2 availability for SP binding was similar for these cell types. However, ACE2 (αACE2-488) co-localization with SP-555 (merged cellular binding-yellow areas) was 50% of SP-555 binding for the hCMEC/D3 cells, compared to that of 15 and 20% for the BHVP and HBVSMC, respectively (Figure 3D). Thus, there were more SP-555 cellular binding to other sites associated with the BHVP and HBVSMC compared to that of the hCMEC/D3 cells (Figures 3A–C). To further elaborate on this, we used excess unlabeled ligands, which will displace the specific binding of the labeled molecule. Excess unlabeled αACE2 (60 μg/ml) reduced SP-555 uptake by about 40–50% (about 50–60% SP-cellular binding remaining) for the hCMEC/D3, BHVP and HBVSMC cells (Figure 3E), which confirmed the data on SP-555 colocalization with αACE2-488 (Figure 3D). In the presence of excess unlabeled SP (1 μM) there was about 40% (30–50%) SP-555 cellular binding still associated with these cell types (Figure 3F). Thus, unlabeled SP displaced the bound labeled SP (SP-555) uptake by 50–70% for these cell types (Figure 3F). SP-555 uptake in the presence of excess unlabeled SP is due to membrane bound and/or non-specific uptake. To establish the levels of extracellular receptor binding, SP-555 uptake was determined at 4°C and compared to that at 37°C. At 37°C, SP-555 uptake was 2.2- to 5.5-fold greater than that at 4°C for these cell type (Figure 3G). Thus, there was likely greater SP interaction with the cells and internalization (including membrane bound) at 37°C. Plasma membrane lipid homeoviscosity is altered by temperature, which could affect protein uptake by the lipid bilayer environment. An illustration diagram of SP-555 uptake *via* ACE2 is shown in Figure 3H. There was no significant effect of angiotensin II, the endogenous ligand of ACE2, on SP uptake by the cerebrovascular endothelial cells (Supplementary Figure 4).

### Sialic acid/GM1-mediates SARS-CoV-2/SP uptake in cerebrovascular cells

**FIGURE 4:**
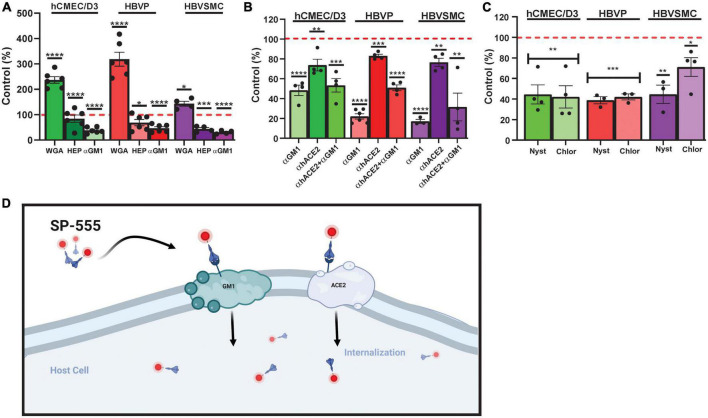
Sialic acid-/GM1-mediated SP uptake by cerebrovascular cells. **(A)** Effect of wheat germ agglutinin (WGA), heparin (HEP) and anti-mono-sialotetra- hexasylganglioside antibody (αGM1) on SP-555 uptake. **(B)** Effect of αGM1, anti-human ACE2 antibody (αACE2) and both αGM1 and αACE2 together on SP-555 uptake. **(C)** Effect of nystatin and chlorpromazine on SP-555 uptake. Controls were SP-555 intensities in the absence of the test compounds. **(D)** Schematic diagram showing a proposed SARS-CoV-2/SP uptake by both GM1 and ACE2. Both GM1 and ACE2 are present within the lipid raft region of cell membranes. Created by using BioRender.com. Red dashed line is the control levels (100%). Values are mean ± SEM. *N* = each data point is a well. Supplementary Figure 4. Statistically analyzed was by analysis of variance (ANOVA) followed by Tukey *post-hoc* test. *P < 0.05, **P < 0.01, and ***P < 0.001 and ****P < 0.0001. GraphPad Prism version 9.2.0 was used.

Glycans, are carbohydrates based polysaccharides that are attached to molecules on the cell surface, and can bind many toxins and pathogens, including viruses (Lingwood, 2011). Sialic acid containing glycan (N-acetyl D-glucosamine) has been reported to play a role in SP binding (Rhea et al., 2021). Similarly, glycosaminoglycans (GAGs), which are sulfate polysaccharides, are thought to play a role in infections (Dick and Vogt, 2014; Aquino and Park, 2016; Lima et al., 2017; Shi et al., 2021). While wheat germ agglutinin (WGA), a lectin that binds sialic acid, increased SP-555 uptake by all three cell types, it was 2.4- to 3.2-greater for hCMEC/D3 and HBVP and 1.4-fold greater for HBVMC compared to that of controls without WGA (Figure 4A). SP uptake and internalization were increased with WGA (Supplementary Figure 5). In contrast, heparin, a polysaccharide, which belongs to the GAG family, did not affect SP uptake in the hCMEC/D3 cells but reduced its uptake to by 30–60% for HBVP and HBVSMC (Figure 4A). Further studies are needed to elaborate on the significance of these findings. Anti- GM1 antibody (αGM1) reduced SP-555 uptake by 60–80% of controls in all of these cell types (Figure 4A). However, in the presence of both antibodies (αACE2 and αGM1) the uptake was similar to that of αGM1 alone (Figure 4B). Since GM1 is present mainly in the lipid raft, SP binding by these cell types could be mediated *via* the caveolin/lipid raft. Further work is needed. There are reports that SP can be taken up into cells by clathrin-mediated endocytosis and by binding to sialic acid residues on cell membrane bound glycoproteins (Lingwood, 2011; Bayati et al., 2021; Rhea et al., 2021). We confirmed that these cell types, including the endothelial cells, can take up transferrin (Supplementary Figure 6A), a molecule that is known to be transported by clathrin-mediated vesicles (Inoue et al., 2007; Mayle et al., 2012). Bovine serum albumin (BSA) conjugated to lactosylceramide BODIPY, a molecule that is involved in the syntheses of gangliosides, including GM1 but not GM4, and taken up by the lipid raft, was taken up by these cell types and colocalized with SP-555 (Supplementary Figure 6B). Thus, it is possible that SP could be taken up by the lipid raft. GM1-BODIPY was also incorporated within the cell membrane (Supplementary Figure 6C). SP-555 is mainly cell membrane bound before possible internalization. While there is no specific inhibitor for each of these uptake mechanisms, nystatin (an inhibitor of lipid raft-mediated uptake) and chlorpromazine (an inhibitor of clathrin-mediated uptake) were tested (Plummer and Manchester, 2013; Wang et al., 2016; Sui et al., 2017). Both inhibitors were effective in blocking SP-555 uptake in each of these cell types by about 60%, except for chlorpromazine in the HBVSMC, which inhibited the uptake by 30% (Figure 4C). It’s tempting to speculate that SP uptake by GM1 assists in its binding to ACE2 (Figure 4B), since both anti-ACE2 and anti-GM1 antibodies elicited the same effect as anti-GM1 alone, and ACE2 and GM1 are located within the caveolin/lipid raft (Glende et al., 2008; Lu et al., 2008; Garofalo et al., 2021). Gangliosides, which are present mostly in the lipid rafts of cell membranes mediate SARS-CoV-2 uptake by facilitating binding to ACE2 (Pirone et al., 2020; Fantini et al., 2020, 2021; Sun, 2021; Nguyen L. et al., 2022). The anti-GM1 antibody (Abcam, cat#Ab 23943), used in our studies was a rabbit polyclonal IgG isoform specific for GM1, with little interaction with the other ganglioside (sialic containing glycosphingolipid), a class of anionic glycosphingolipids (manufacturer information). Thus, there is little, if any, interaction of this anti-GM1 with GM2 and GM3. In addition, there was > 70% inhibition of SP uptake with this anti-GM1 antibody (Figure 4), and thus, < 30% of the SP uptake could be due to other facilitators.

### Higher mutant SARS-CoV-2/SP uptake by cerebrovascular cells

**FIGURE 5:**
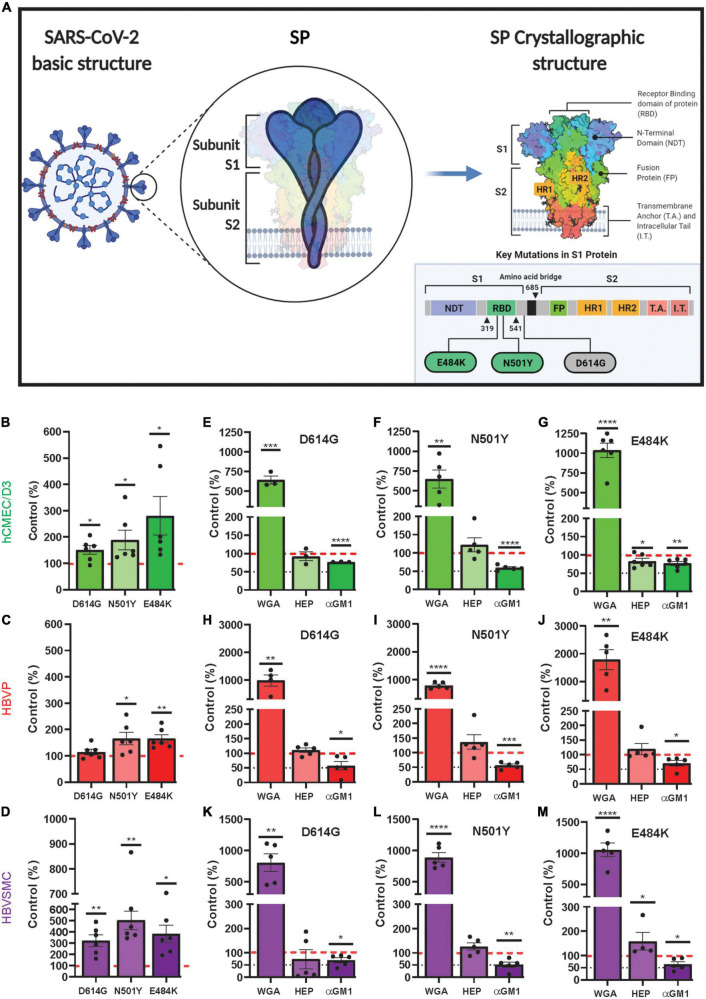
Increased mutant SARS-CoV-2/SP uptake by cerebrovascular cells. **(A)** Schematic diagram showing the mutation sites of the three mutants (D614G, N501Y and E484K) used in this study. Adapted from “An In-depth look into the Structure of the SARS-CoV2 Spike Glycoprotein,” by (2021). Retrieved from https://app.biorender.com/biorender-templates. **(B–D)** Mutant SP-555 uptake compared to control wild-type SP (red dashed line) for hCMEC/D3 **(B)**, HBVP **(C)**, and HBVSMC **(D)**. **(E–M)** The effects of wheat germ agglutinin (WGA), heparin and anti GM1 (a mono sialic acid ganglioside) on the three cell types, hCMEC/D3 **(E–G)**, HBVP **(H–J)**, and HBVSMC **(K–M)**. Controls are the SARS-CoV-2/SP uptake without the test compounds. Values are mean ± SEM. *N* = data points (wells) shown with the histogram. Red dashed line is the control levels (100%). Supplementary Figure 6.

While SARS-CoV-2 variants harbor many mutation sites, three mutation sites were selected from variants of concern (WHO Coronavirus (COVID-19) Dashboard, n.d.) to determine if their uptake is altered in these cell type (Table 1). These mutations were within the RBD (N501Y and E484K) and one (D614G), a common mutation site, which is outside the RBD and furin cleavage site (Figure 5A). For the hCMEC/D3 cells, the uptake of mutants D614G, N501Y and E484K was significantly increased by 1. 5-, 1. 9-, and 2.8-fold, respectively, compared to control wild type SP (Figure 5B). In contrast, for the HBVP, the uptake of mutant D614G was unchanged, but for N501Y and E484K it was significantly increased by 1.7-fold compared to that of controls (Figure 5C). However, for the HBVSMC, the uptake of mutants D614G, N501Y, and E484K was significantly increased by 3. 2-, 5. 0-, and 3.8-fold, respectively, compared to that of controls (Figure 5D). Thus, again, there was differential mutant SP uptake by the three cell types. Uptakes of D614G, N501Y, and E484K were highest for the HBVSMC, but there was no significant differences for the hCMEC/D3 and HBVP (Supplementary Figure 7). This may reflect the type and distribution of SP receptors. Mutation sites E484K and N501Y confer gain-of-function, and N501Y increases SP affinity (Bayarri-Olmos et al., 2021; Tian et al., 2021; Xie et al., 2021). Mutant E484K also confers immune escape (Weisblum et al., 2020; Harvey et al., 2021). Mutant D614G seem to confers increased infectivity and transmissibility (Volz et al., 2021).

**TABLE 1:** Main Characteristics of SARS-CoV-2 variants containing the SP mutation sites used in this study.

Variant of concern		Alpha	Beta	Gamma	Delta	Omicron
Pango lineage(s)		B.1.1.7	B.1.351	P.1	B.1.617.2	B.1.1.529.1
			B.1.351.2	P.1.1	AY. (1–12)	B.1.1.529.2
			B.1.351.3	P.1.2		B.1.1.529.3
Origin (date)		UK (December 2020)	S. Africa (December 2020)	Brazil (November 2020)	India (October 2020)	S. Africa/Botswana (November 2021)
Critical mutation	RBD	N501Y	K417N, E484K, N501Y	K417T, E484K, N501Y	T478K, E484K, N501Y	E484A, T478K, N501Y
	Other	D614G, P681H	D614G	D614G	D614G, P681R	D614G, P681H, N679K
Main properties		-50% Increased transmission	-50% increased transmission	-Significantly reduced susceptibility to bamlanivimab and etesevimab treatments	-Increased transmissibility	-Increased transmissibility
		-Potential increase severity	-Reduced susceptibility to bamlanivimab and etesevimab	-Reduction neutralization by convalescent and post-vaccination sera	-Potential reduction in neutralization by some antibody treatments	-Reduced neutralization by some antibody treatments
		-Minimal impact on neutralization by convalescent and post-vaccination sera	-Reduced neutralization by convalescent and post-vaccination sera	-Significantly increased severity	-Potential reduction by post-vaccination sera	-Less severe
			-Increased severity		-Increased severity	

**FIGURE 6:**
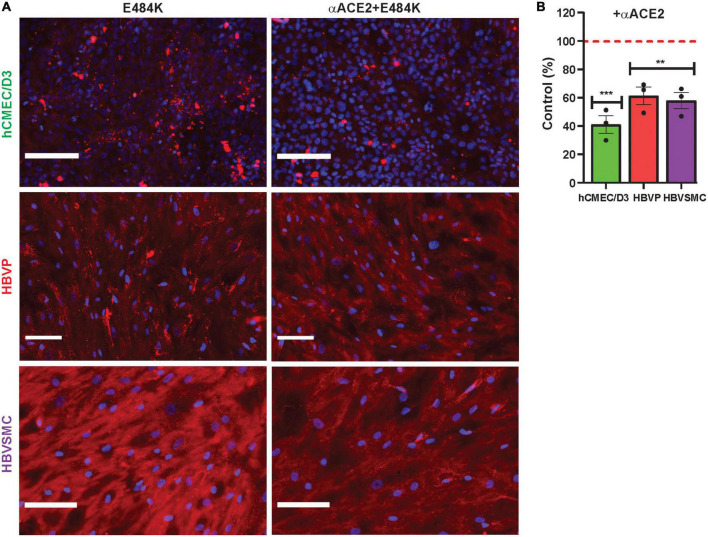
Anti-ACE2 suppressed mutant SARS-CoV-2/SP E484K uptake by cerebrovascular cells. **(A,B)** Excess unlabeled αACE2 suppressed labeled SP-555 E484K uptake in the hCMEC/D3 cells, HBVP, and HBVSMC compared to controls in the absence of unlabeled αACE2 for each cell type. Red dashed line is control levels in the absence of αACE2 (100%). Values are mean ± SEM. *N* = number of data points (wells) shown with the histogram. Scale bar = 100 μm. Supplementary Figure 7. **P < 0.01; ***P < 0.001.

Mutant SP uptake was considerably increased with WGA by 6.9–17.9-fold in the three cell types compared to their respective mutant control without WGA (Figures 5E–M). This was 2.9–7.7-fold greater than that seen for WGA with the wild type SP (Figures 4A–C). Mutant SP uptake was inhibited by anti-GM1 in the three cell types (Figures 5E–M). In contrast, heparin had no effect except for E484K uptake in hCMEC/D3 and HBVSMC, which was decreased and increased, respectively (Figures 5E–M). Heparin inhibits SP binding in non-cell-based assays, as reported (Gupta et al., 2021; Ali et al., 2022). While It is unclear on how heparin increased E484K uptake in HBVSMC, it is possible that heparin binds E484K and this complex increased its binding to ACE2, a reported mechanism (Ali et al., 2022). Further work is needed. The neutralization effect of anti-GM1 antibody was less effective (Figures 5B–M) by 1.5–3.1-fold compared to wild type SP (Figures 4A–C). These differences may be due to the effectiveness of the mutants SP binding and the distribution/accessibility of the glycans and ACE2 on the three cell types. Excess αACE2 (60 μg/ml) suppressed SP E484K uptake (Figures 6A, B) but this was also less effective compared to that of wild type SP with the exception of hCMEC/D3 cells (Figures 3A–G).

## Discussion

We have shown that there was differential uptake of SARS-CoV-2/SP by three human cerebrovascular cell types, endothelial cells, pericytes, and vascular smooth muscle cells. The endothelial cells, the physical site of the blood brain barrier (BBB), had the lowest capacity to take up SARS-CoV-2/SP compared to that of the other cell types, which may explain the low viral replication by the cerebrovasculature (Constant et al., 2021). In addition, SARS-CoV-2/SP uptake was dependent on the duration of its exposure and on its concentration (titer). Thus, longer exposure to SARS-CoV-2 and a higher titer, which are variable in the pathogenesis of the disease, will contribute to COVID-19 severity and possibly its neurological manifestations. SARS-CoV-2/SP uptake was mediated by both ACE2 and GM1, which contains sialic acid, but these two processes were not independent.

Uptake of SARS-CoV-2/mutant SP, N501Y, E484K, and D614G, were increased by the three cell types compared to that of the wild type SAR-CoV-2/SP, except for D613G in pericyte, which was unchanged. While WGA considerable increased the uptake of the three mutants SARS-CoV-2/SP, anti-ACE2 and anti-GM1 were less effective in neutralizing mutant SARS-CoV-2/SP uptake compared to that of the wild type controls. There was differential SAR-CoV-2/mutant SP uptake by these cell types. Therefore, in addition to ACE2, the established SARS-CoV-2 receptor, glycolipids, especially ganglioside, is a SARS-CoV-2/SP facilitators. While our hypothesis was that ACE2 will be the main facilitator, our data show that SARS-CoV-2 interaction with these host cells was more complex. It appears that multiple facilitators are involved to ensure that the virus access host cell machinery for its survival.

Our data show that ACE2 is a facilitator of wild type and mutant SARS-CoV-2 uptake by cerebrovascular cells, as it is for other host cell. There is an extensive literature on this (Pirone et al., 2020; Qiao et al., 2020; Brady et al., 2021; McQuaid et al., 2021). Even though each cell type expressed ACE2, there is differential effects by the cell type that can be subtle. Almost twice as much of SARS-CoV-2/SP colocalized with ACE2 for the endothelial cells compared to that of pericytes and vascular smooth muscle cells. Despite this the endothelial cells restricted the total uptake of SARS-CoV-2/SP compared to the other two cell types. The higher uptakes of the mutant SARS-CoV-2/SP (E484K, N501Y and D614G) likely associated with increased binding to facilitators, including ACE2 (WHO Coronavirus (COVID-19) Dashboard, n.d.). Further work is needed to explain why SARS-CoV-2 mutant D614G uptake is unchanged in the HBVP (human pericyte).

In contrast to ACE2, the role of gangliosides, sialic acid containing glycans and possibly other glycolipids in SARS-CoV-2 uptake is emerging as another viral facilitator. Glycolipids are a large group of heterogenous compounds, which consist of monosaccharide residues (head group on mainly the outer cell membrane surface) linked by a glycosidic bond to a hydrophobic lipid moiety (within the cell membrane), such as acylglycerol, sphingoid, or ceramide (Lingwood, 2011; Malhotra, 2012; Sipione et al., 2020). The sphingoids or glycosphingolipids are found mainly in animals cell membranes, and include neutral, such as cerebrosides, and acidic molecules, such as gangliosides. Gangliosides consist of a ceremide lipid moiety linked to an oligosaccharide chain of hexoses and sialic acids. Gangliosides have 0,1, 2, or 3 sialic acid moiety, and GM1, which has one sialic acid residue, is a common member of the ganglioside group (Lingwood, 2011; Malhotra, 2012; Sipione et al., 2020). They are mainly present in the lipid raft of plasma membrane (Brown and London, 2000; Degroote et al., 2004; Moreno-Altamirano et al., 2007; Lingwood, 2011; D’Angelo et al., 2013). While these molecules are ubiquitous expressed in cell membranes, studies on the relative abundance of each glycolipid or ganglioside and their functions in different cell types have not been reported (Sipione et al., 2020). This is likely due to the heterogeneity of these diverse molecules, low levels to detect and their varied functions (Svennerholm, 1963; Sipione et al., 2020). While it is believed that glycolipids make up a small fraction of the plasma membrane lipids, they have essential functions, which include plasma membrane fluidity, stabilization of the plasma membrane, protein receptor distribution, protein-protein interaction, ligand-receptor interaction, cell-cell communications, adhesion and release of neurotrophins, due mainly to their amphipathic nature (Battistin et al., 1985; Vorbrodt, 1986; Iwabuchi et al., 1998; Hakomori, 2000; Mitsuda et al., 2002; Jeyakumar et al., 2003; Ngamukote et al., 2007; Furian et al., 2008; Lingwood, 2011; Malhotra, 2012; Sipione et al., 2020).

Gangliosides, including GM1, are expressed in vascular endothelial cells, and involved in endocytosis and signaling (Born and Palinski, 1985; Weigel and Yik, 2002). Brain micro vessels and cultured brain endothelial cells, including cell lines, express sialic acid oligosaccharides (N acetyl-D-glucosamine), which binds WGA (Fatehi et al., 1987; Plattner et al., 2010). SARS-CoV-2/SP binds to WGA and increased its uptake into brain, in mice (Erickson et al., 2021; Rhea et al., 2021). Our data confirm that WGA binds SARS-CoV-2/SP, but it’s mainly located on the endothelial cell surface. However, our data show that anti-GM1 antibody suppressed SARS-CoV-2/SP uptake in all three cerebrovascular cell type, and thus, a facilitator for SARS-CoV-2/SP. Human brain endothelial cells contain low levels of gangliosides, including GM3, GM2 and GM1 (Gillard et al., 1987; Kanda et al., 1994; Duvar et al., 1997, 2000; Müthing et al., 1999). These compounds are structurally and functionally polymorphic and the content varies in different tissues, age, conditions and animals (Ledeen and Wu, 2015; Schengrund, 2015; Aureli et al., 2016).

However, GM1 protects the cerebrovasculature from photochemical-induced (rose Bengal)-induced damage (Frontczak-Baniewicz et al., 2000), blast traumatic brain injury (Rubovitch et al., 2017), oxidative damage (Zhao et al., 2015). neurovascular injury of glutamate and kanate (Favaron et al., 1988), alcohol injury (Hungund et al., 1990), calcium toxicity (Nakamura et al., 1992), injury caused by middle cerebral artery occlusion (MCAO) (Zhang et al., 2019), and diabetic injuries (Figliomeni et al., 1992). GM1 also increases cerebral blood flow (CBF) mediated by nitric oxide (Furian et al., 2008). In addition, GM1 and GM2 increase cell proliferation, DNA synthesis and protects the VSMC (Sachinidis et al., 2000; Gouni-Berthold et al., 2001). Gangliosides (GM2 > GM1) potentiates platelet-derived growth factor (PDGF) induced proliferation of VSMC (Sachinidis et al., 1996; Sasaki and Toyoda, 2019). GM1 functions as a co-receptor for fibroblast growth factor in endothelial cells (Rusnati et al., 2002, 1999). Thus, it is possible that SARS-CoV-2 interaction at the cerebravasculature could contribute to cerebrovascular dysfunction and possibly Alzheimer’s disease (AD)-like symptoms.

The brain contains many forms of gangliosides, but about 95% of the total is made up of GM1, GD1a, GD1b, and GQ1b (Gong et al., 2002; Kaida et al., 2009; Wang and Yu, 2013; Chiricozzi et al., 2020). Levels of GM1 are reduced with aging and in AD, while GM2 is increased (Krauss and Burke, 1982; Kracun et al., 1991; Svennerholm et al., 2002; Svennerholm and Gottfries, 2008; Liu et al., 2015; Ledeen and Wu, 2018). GM1 levels are also reduced in other neurodegenerative diseases, such as Huntington’s and Parkinson’s diseases (Svennerholm et al., 2002; Desplats et al., 2007; Schneider et al., 2010; Magistretti et al., 2019; Sipione et al., 2020). GM1 levels in CSF has been shown to improve day-to-day activity in AD (Augustinsson et al., 1997), and it increases choline acetyl esterase activity (ChAT) (Fong et al., 1995). It potentiates the effects of neurotrophic factor (Facci et al., 1990; Ferrari et al., 1993; Garofalo and Cuello, 1994) and basic fibroblast growth factor (Iwashita et al., 1996). GM1 has been shown to reduced amyloid-β toxicity (Kreutz et al., 2011). Thus, SARS-CoV-2 interacting with gangliosides may lead to AD-like symptoms.

However, glycolipids are known to bind many toxins and pathogens, including viruses (Lingwood, 2011; Sipione et al., 2020; Nicoli et al., 2021). GM1 is a receptor for microbes. It binds toxins, such as cholera and Shiga, and viruses, such as influenza and HIV (Sachinidis et al., 2000; Ilver et al., 2003; Suzuki, 2005; Lehmann et al., 2006; Chinnapen et al., 2007; Varki, 2007; Johannes, 2017; Chiricozzi et al., 2020; Cutillo et al., 2020). Sialic acid on GM1 binds the B subunit of cholera toxin and affluenza A (Wu et al., 2007). Guillain-Barre syndrome (GBS) appears to be associated with the presence of anti-gangliosides antibodies in blood, and there were reports of GBS-like effects in some SARS-CoV-2 and influenza vaccinated subjects (Kaida et al., 2009; Vellozzi et al., 2014; Martín Arias et al., 2015; Lunn et al., 2021; Shapiro Ben David et al., 2021). However, sialic containing glycans, like GM1, offers an additions potential target to be considered and to explore therapeutics for of SARS-CoV-2.

## Conclusion

SARS-CoV-2/SP uptake by three cell types of human cerebrovasculature (endothelial cells, pericytes, and VSMC) was time and concentration/titer dependent. It was the lowest for the endothelial cells, which may limit viral uptake in the normal healthy brains. Wild type SARS-CoV-2/SP, and mutant SARS-CoV-2/SP containing the common mutation sites, D614G, N501Y, and E484K, as seen in variants of interest, were differentially taken up by GM1- and ACE2-mediated mechanisms by the three the cell types. WGA, a lectin that binds sialic acid, increases mutant SARS-CoV-2/SP considerably compared to that of the wild type controls. While the mutant SARS-CoV-2/SPs were more effective in binding, anti-ACE2 and anti-GM1 antibodies were less effective in neutralizing their uptake. In general, the uptake mechanisms were similar in each cell type. Since SARS-CoV-2 uptake is the initial step in the viral penetration into cells, the data suggest that GM1, which contains one sialic acid residue, is also an important entry point of SARS-CoV-2 into these cells. GM1 could be an addition potential SARS-CoV-2 and therapeutic target at the cerebrovasculature, and perhaps other cell types. It is likely that in the severely infected patients there is greater uptake by the brain due to the degree of cerebrovasculature dysfunction in the aging brain, prior health conditions, complication with the infections, and the titre and duration of the viral exposure. Cardio-respiratory failure is likely a major contributed to the neurological symptoms since the brain is dependent on an adequate supply oxygenated blood containing nutrients, especially glucose.

## Limitations of the study

This is an *in vitro* study designed to explore possible mechanisms for SARS-CoV-2/SP uptake by three cell types of the cerebrovasculature exposed to the same titer/concentration for 4 h (usually) in controlled conditions. In human, it is likely rare to be exposed to the same viral titer for 4 h. The RBD of the SARS-CoV-2/SPs was used as a model of SARS-CoV-2, since it is essential for viral entry into host cells and viral binding/uptake was investigated in this study. Thus, only the attachment part of the viral life cycle can be explored with. Also, other cell types of the neurovascular unit, such as astrocytes and microglia need to be explored to confirm SARS-CoV-2/SP uptake mechanisms. However, it is likely that there will be similar mechanisms since these cells also express ACE2 and have sialic containing glycans, including GM1.

## Further studies

There are a number of possible lines for future studies, which include the following:

1. The mechanisms and relevance of SARS-CoV-2/SP interaction to gangliosides and possibly other plasma membrane glycolipids.
2. Is there cell-type differences in levels of gangliosides and glycolipids in the aging cells and does it affects SARS-CoV-2 uptake?
3. While SARS-CoV-2/SP uptake at the cell surface leads to its cellular internalization, it could also interaction with other receptors in the lipid raft, which would elicit deleterious responses by the endothelial cells and other cell types. The role of gangliosides/glycolipids is this process needs to be explored.
4. One of the added value of *in vitro* cellular studies is to explore possible mechanisms in controlled conditions. However, it is important to perform *in vivo* studies using approached as close as possible to that of the *in vitro* conditions to establish if similar outcomes are possible.
5. Uptake of SARS-CoV-2/SP from blood by the endothelium will be limited by its rapid clearance from the blood compartment, and thus, a high titer may not be easily maintained for a long duration. In contrast, for the nasal cavity and respiratory surfaces, it is possible to have a higher viral titer for a longer duration. SARS-CoV-2 causes a severe respiratory infection (COVID-19), which is spread mainly by breathing in air-borne droplets containing the virus, and thus, should have a greater effect on the delicate respiratory membrane of the lungs. Nevertheless, SAS-CoV-2/SP uptake from blood is relevant since the virus can escape into blood from the damaged thin respiratory membrane. Once in blood the virus is able to access the vasculature endothelial cells, including that at the cerebrovasculature. It would be useful to compare uptake of SARS-CoV-2/SP at these two sites and to determine whether it is influenced by levels of gangliosides, other glycolipids and ACE2.

## Data availability statement

The original contributions presented in this study are included in the article/Supplementary material, further inquiries can be directed to the corresponding author.

## Author contributions

CM performed experiments, imaging, preparation of figures and table, and contributed to manuscript preparation. AS performed experiments, imaging, data analysis, preparation of figures and table, contributed to manuscript preparation, and prepared the references. ID contributed resources and critical review of the manuscript. RD provided the concept, designed the study, and wrote the manuscript. All authors approved the manuscript.
