# Supplementary material for: Uptake of severe acute respiratory syndrome coronavirus 2 spike protein mediated by angiotensin converting enzyme 2 and ganglioside in human cerebrovascular cells
Source: Front Neurosci. 2023 Feb 16;17:1117845. doi: 10.3389/fnins.2023.1117845 (PMC9980911; doi:10.3389/fnins.2023.1117845)
Supplement: Supplementary file 1 [file Presentation_1.pdf]

Uptake of severe acute respiratory syndrome coronavirus 2 Spike protein mediated by angiotensin converting enzyme 2 and ganglioside in human cerebrovascular cells

Neurovascular uptake of spike proteins

Authors: Conor McQuaid, Alexander Solorzano, Ian Dickerson, Rashid Deane *

Del Monte Institute Neuroscience, Department of Neuroscience, University of Rochester, URMC, 601 Elmwood Avenue, Rochester, NY 14642, USA

***Corresponding author**: Email address: Rashid_Deane@urmc.rochester.edu

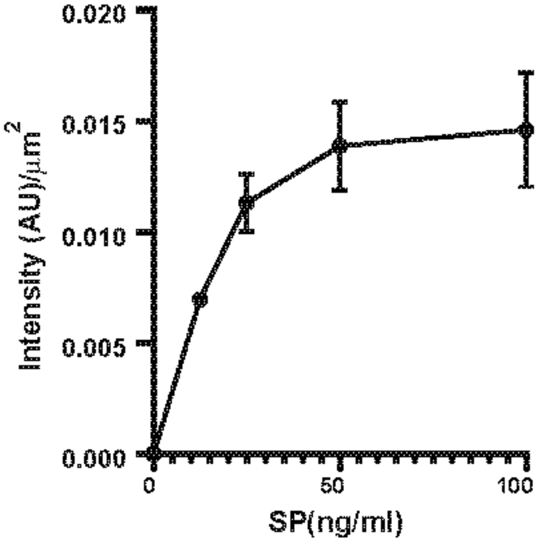

Supplementary Figure 1. Human ACE2 binds SP555 in a non-cell-based assay. Recombinant human ACE2 dissolved in carbonate/bicarbonate buffer was immobilized (2 μg/ml) on glass slides for 1 hr at room temperature (RT), blocked with a non-protein buffer (Pierce Blocking buffer), washed, incubated with SP-555 at different concentrations in HBSS for 1 hr at RT, washed, mounted and imaged. SP-555 intensity from 10 fields for

each concentration were analyzed and expressed as intensity/μm^2. This is a non-cell-based assay. Values are mean ± SEM.

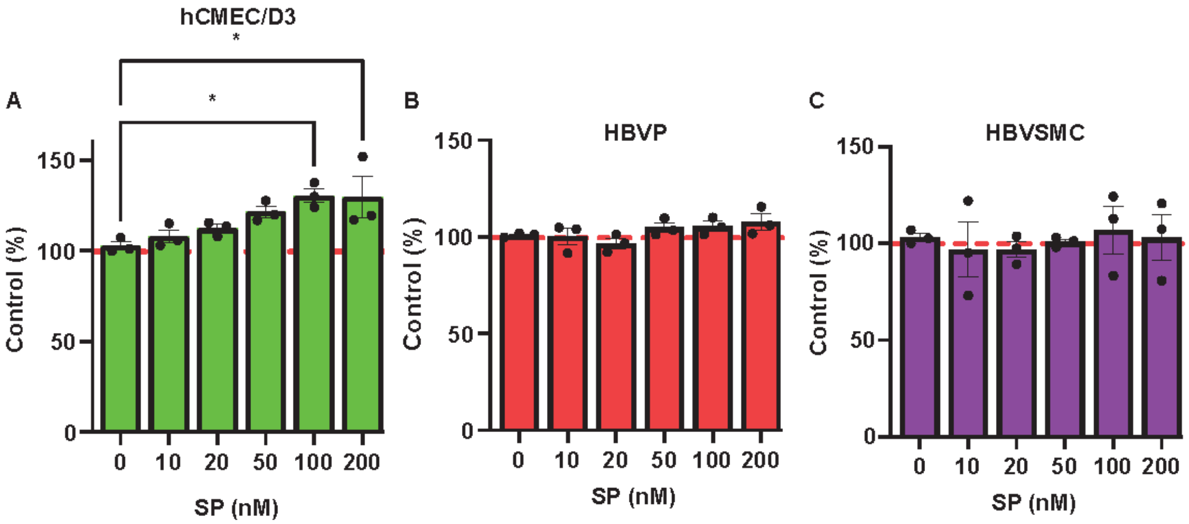

Supplementary Figure 2. **SP not toxic to these cerebrovascular cell types. A-C**. Cell viability determined by using the MTT cytotoxicity assay for the hCMEC/D3 (**A**), HBVP (**B**) and HBVSMC (**C**). Red dashed line is control levels without SP(100%). Values are mean ± SEM. N=3 wells per group. Statistically analyzed was by analysis of variance (ANOVA) followed by Tukey post hoc test. *$P < 0.05$. GraphPad Prism version 9.2.0 was used.

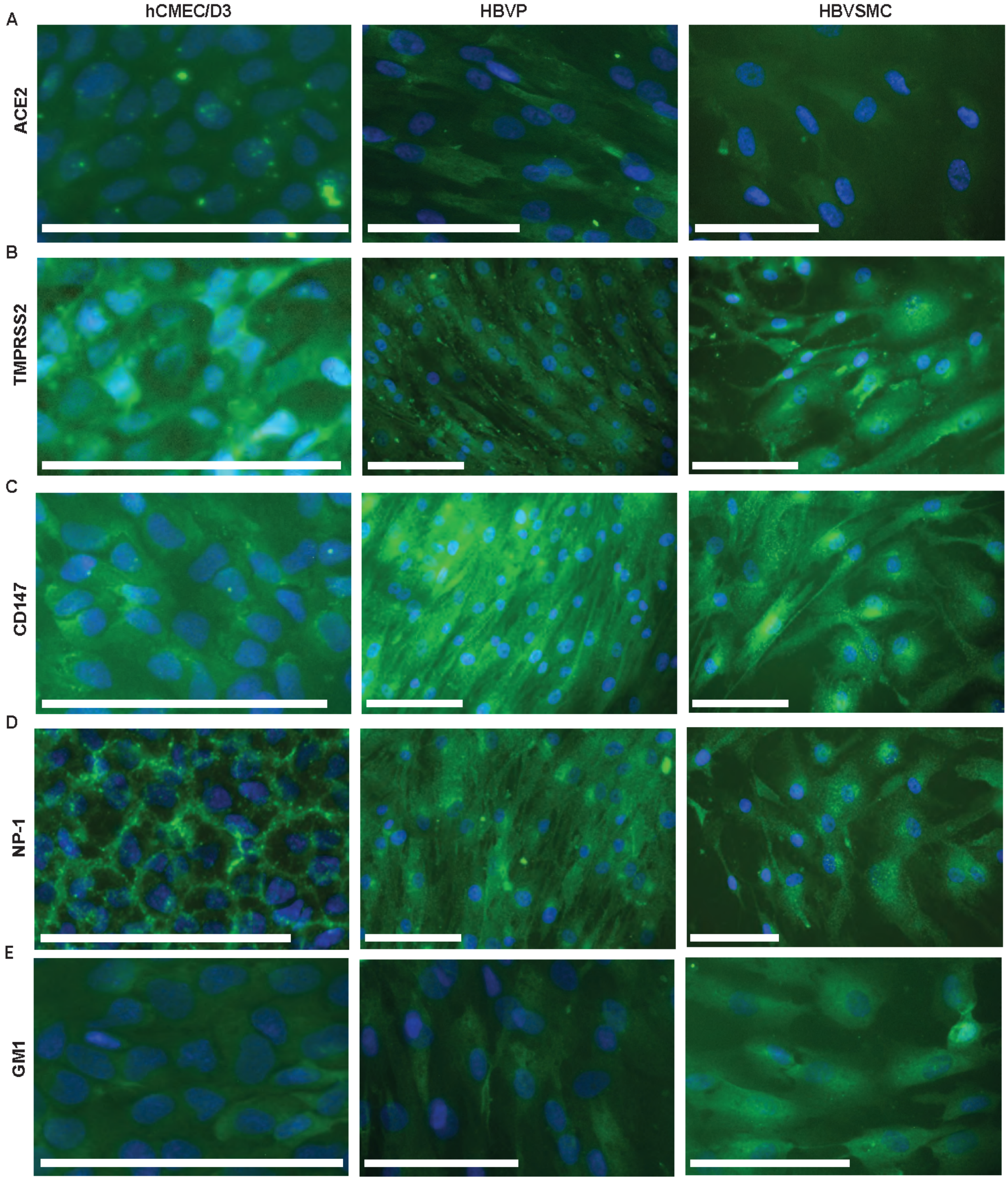

Supplementary Figure 3. SARS-CoV-2 associated receptors present on these cerebrovascular cells. **A-E**. Representative images (green) confirming the presence of **A**) angiotensin converting enzyme 2 receptor (ACE2), **B**) transmembrane serine protease

2(TMPRSS2), **C**) extracellular matrix metalloprotease inducer (CD147), **D**), neuropilin-1 (NP-1), and **E**) ganglioside (GM1) on these cerebrovascular cell types (hCMEC/D3, HBVP and HBVSMC). Blue (DAPI) is the cell nucleus. Scale bar =50 μm.

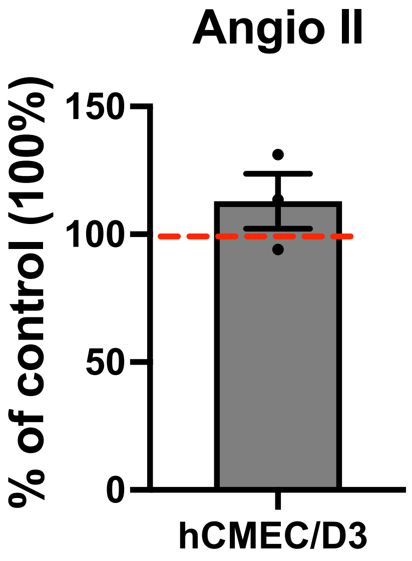

Supplementary Figure 4. No effect of angiotensin II on SP uptake by the hCMEC/D3 cells. SP uptake in the presence of angiotensin II (Angio II) by the cerebrovascular endothelial cells (hCMEC/D3). Values are mean ± SEM. N= number of data points (wells) shown with the histogram. Controls are SP uptake in the absence of angiotensin II. Red dashed line is the control SP uptake (100%).

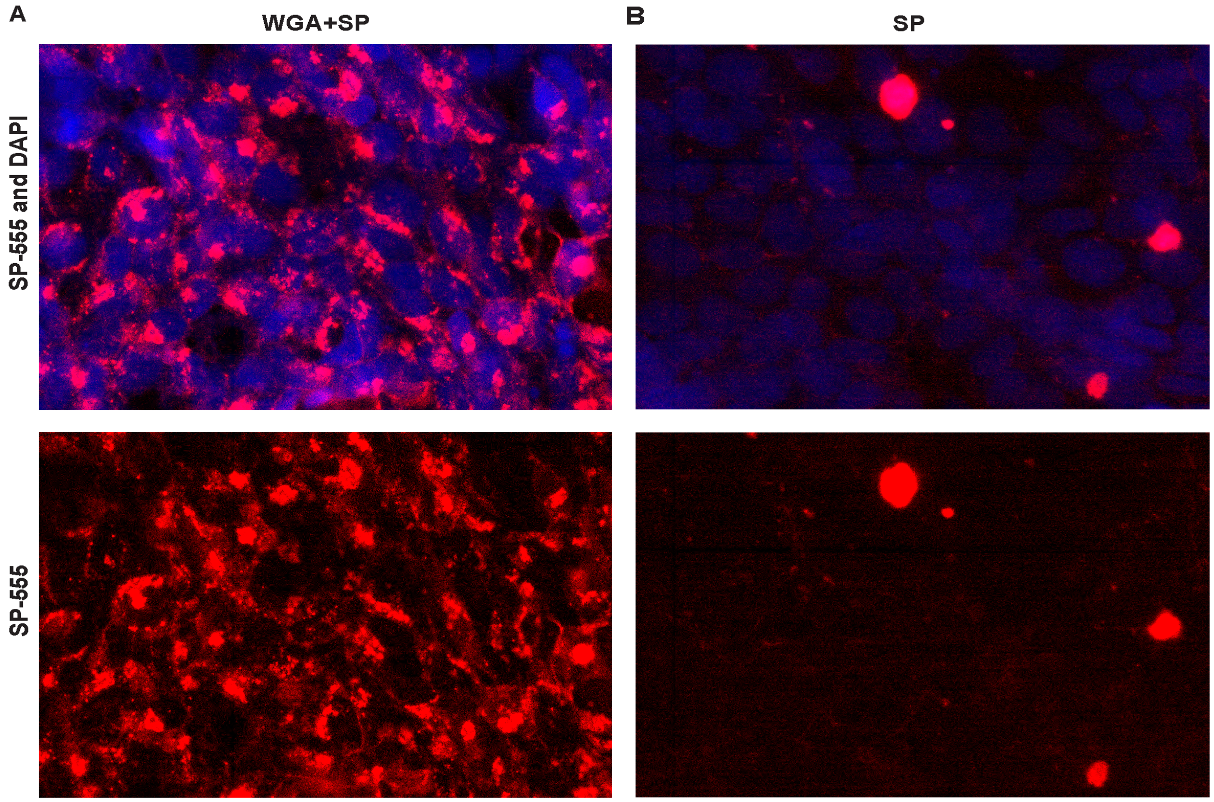

Supplementary Figure 5. Wheat germ agglutinin increases SP uptake. Representative image of SP-555 uptake with (**A**) and without (**B**) wheat germ agglutinin (WGA) for hCMEC/D3. Scale bar =100 μm.

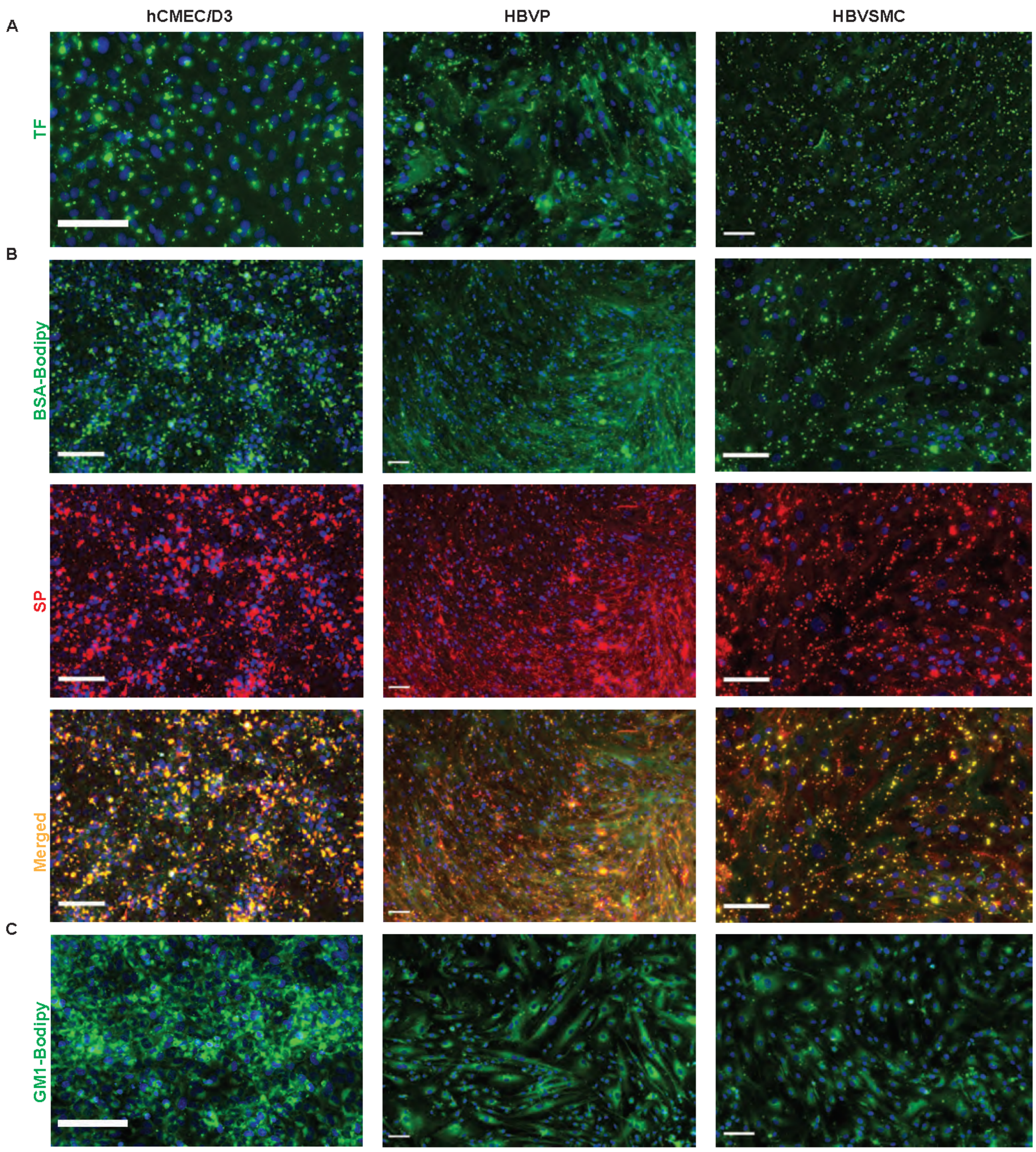

Supplementary Figure 6. Transferrin and BODIPY (BSA-BODIPY) uptake in these cerebrovascular cells. **A.** Representative images confirming uptake of transferrin (TF-488). **B**. Representative images showing bovine serum albumin (BSA) conjugated to

lactosylceramide BODIPY (BSA-BODIPY) uptake and its colocalization with SP-555. **C**. GM1-BODIPY uptake by these cells. Scale bar =100 μm.

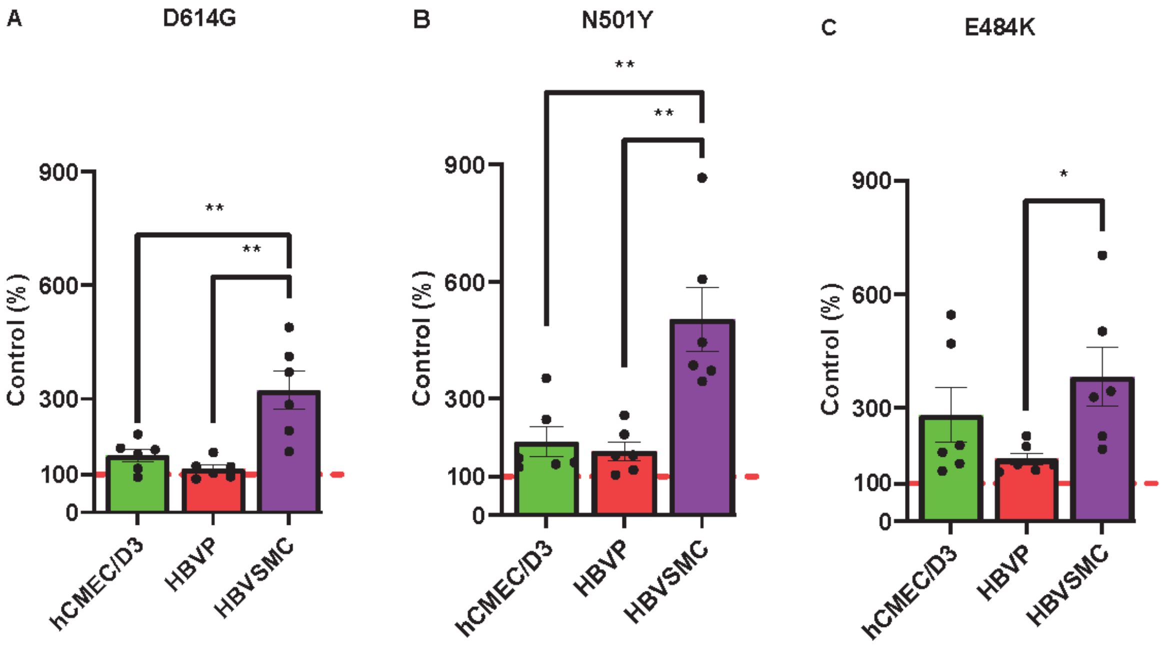

Supplementary Figure 7. Increased mutant SP uptake by the cerebrovascular cells. A-C. Comparison of the mutant SP uptake between the three cell types (hCMEC/D3, HBVP and HBVSMC for D614G (**A**), N501Y(**B**) and E484K (**C**). Values are mean ± SEM. N= number of data points (wells) shown with each histogram. Red dashed line is the control levels with wild type SP(100%). Statistically analyzed was by analysis of variance (ANOVA) followed by Tukey post hoc test. *$P < 0.05$ and **$P < 0.01$. GraphPad Prism version 9.2.0 was used.
